# Necessity and time interval of regional lymph node dissection for malignant melanoma with positive sentinel lymph node biopsy: Does the time interval between sentinel lymph node biopsy and completion of lymph node dissection affect outcome in malignant melanoma? A retrospective cohort study – a commentary

**DOI:** 10.1097/JS9.0000000000000409

**Published:** 2023-06-05

**Authors:** Jingxiu Chai, Zubing Mei, Yuchong Chen

**Affiliations:** a Department of Dermatologic Surgery, Shanghai Skin Disease Hospital Affiliated to Tongji University; b Department of Anorectal Surgery, Shuguang Hospital, Shanghai University of Traditional Chinese Medicine, Shanghai, People's Republic of China

Skin melanoma is a malignant tumor that originates from melanocytes in the skin and other organs. In recent years, the incidence rate has been increasing. According to data from the American Joint Committee on Cancer (AJCC), there were 99 780 new cases of melanoma patients and 7650 deaths in the United States in 2022^1^. Skin melanoma has a high degree of malignancy, is prone to metastasis, and has a high mortality rate, which has received increasing attention from researchers in recent years. The prognosis of melanoma patients and the selection of treatment options are closely related to the TNM (tumor, node, and metastasis) staging of the tumor, among which the evaluation of whether there is lymph node metastasis is particularly important. Sentinel lymph node biopsy (SLNB) is an effective measure of evaluating whether there is lymph node metastasis in the regional lymph nodes.

Most patients who found metastasis in SLNB are usually recommended to have immediate completion lymph node dissection (CLND), but recent studies have proposed different opinions. CLND may increase surgical risks, lymphedema, and other complications. Is immediate CLND necessary? If it is performed, does the time interval between metastasis in SLNB and CLND affect the patient’s prognosis?

Richtig *et al*. conducted a single-center retrospective study that reviewed 121 patients diagnosed with malignant melanoma and with a history of metastasis in SLNB, with a median follow-up time of 42 months. The study recorded the Breslow tumor thickness of the primary lesion, the presence of ulceration, the histopathological parameters of the SLNB, the presence of metastasis, the subsequent CLND, and the disease prognosis. They particularly analyzed two-time parameters: the time interval between diagnosis (primary lesion resection) and SLNB and the time interval between metastasis in SLNB and CLND.

The study found that the main risk factors for CLND positivity were the time interval between diagnosis of malignant melanoma and SLNB (*P*=0.0034) and the N stage of TNM staging (*P*=0.0026), while the Breslow tumor thickness of the primary lesion showed no significant correlation (*P*=0.11) and there was no correlation with ulceration or localization of onset^2^.

The question of whether the time interval between metastasis in SLNB and CLND affects prognosis is worthy of consideration by clinical physicians. The retrospective data in this article provides data for clinical decision-making and is helpful for clinical practice. However, this article also has its limitations, such as being a single-center retrospective study without randomization and a relatively small number of enrolled patients. More patients should be included to improve the credibility of the conclusions.

Clinical studies on whether CLND affects the prognosis of melanoma patients have also been discussed worldwide. A multicenter, randomized controlled, phase III trial (DeCOG-SLT) in Germany compared the survival rates of melanoma patients with metastasis in SLNB who underwent CLND or not. The study found that there was no significant difference in survival rates between patients who received CLND treatment and those who did not. The researchers suggested that for melanoma patients with lymph node micrometastases within 1 mm diameter, CLND is not recommended^3^.

In addition, another international, multicenter, phase III trial (MSLT-II) enrolled 1934 melanoma patients and randomly assigned them to the CLND or observation with frequent nodal ultrasonography. During a median follow-up of 43 months, it was found that immediate CLND did not increase melanoma-specific survival in patients with metastasis in SLNB^4^. These findings proposed different opinions on the current clinical decision to perform immediate CLND for patients with metastasis in SLNB.

However, it is worth noting that the decision to replace immediate CLND with high-quality ultrasound surveillance after metastasis in SLNB may not be applicable to all regions. Due to differences in medical and economic conditions, caution should be made in recommending ultrasound follow-up observation for patients with metastasis in SLNB with limited medical follow-up conditions. It is hoped that more clinical research will focus on these issues, which will provide important evidence for clinical decision-making in the future.

## Ethical approval

Not applicable.

## Sources of funding

None.

## Author contribution

J.C.: completed the manuscript; Y.C. and Z.M.: reviewed the manuscript and edited language.

## Conflicts of interest disclosure

There are no conflicts of interest.

## Research registration unique identifying number (UIN)

The paper is a commentary to the Editor.

## Guarantor

Yuchong Chen.

## Provenance and peer review

Commentary, internally reviewed.
